# Supplementary material for: Associations between Adverse Childhood Experiences (ACEs) and Lifetime Experience of Car Crashes and Burns: A Cross-Sectional Study
Source: Int J Environ Res Public Health. 2022 Nov 30;19(23):16036. doi: 10.3390/ijerph192316036 (PMC9735663; doi:10.3390/ijerph192316036)
Supplement: Supplementary file 1 [file ijerph-19-16036-s001.zip › Supplementary file S1 111022.pdf]

Supplementary Materials S1: Questions and qualifying responses for adverse childhood experiences (ACEs) and injury types

	Question *(response options)*	Qualifying response
**ACEs**	All ACE questions were preceded by the statement "While you were growing up, before the age of 18..."	
Physical abuse	How often did a parent or adult in your home ever hit, beat, kick, or physically hurt you in any way? This does not include gentle smacking for punishment. *(never; once; more than once; prefer not to say)*	Once or more than once
Verbal abuse	How often did a parent or adult in your home ever swear at you, insult you, or put you down? *(never; once; more than once; prefer not to say)*	More than once
Sexual abuse	Did an adult or someone at least five years older than you sexually abuse you by touching you or making you undertake any sexual activity with them? *(yes; no; prefer not to say)*	Yes
Parental separation	Were your parents ever separated or divorced? *(yes; no; prefer not to say)*	Yes
Domestic violence	How often did your parents or adults in your home ever slap, hit, kick, punch, or beat each other up? *(never; once; more than once; prefer not to say)*	Once or more than once
Mental illness	Did you live with anyone who was depressed, mentally ill or suicidal? *(yes; no; prefer not to say)*	Yes
Alcohol abuse	Did you live with anyone who was a problem drinker or alcoholic? *(yes; no; prefer not to say)*	Yes
Drug abuse	Did you live with anyone who used illegal street drugs or abused prescription medications? *(yes; no; prefer not to say)*	Yes
Incarceration	Did you live with anyone who served time or was sentenced to serve time in a prison or young offenders' institution? *(yes; no; prefer not to say)*	Yes
**Injuries**	All injury questions were proceeded by the question, "About how many times in your life have you experienced the following…"	
Broken bone	A broken bone? *(never; once; 2-5 times; 6-10 times; more than 10 times; prefer not to say)*	Never, once or 2+ times
Car crash	Been in a car crash, regardless of whose fault it was? *(never; once; 2-5 times; 6-10 times; more than 10 times; prefer not to say)*	Never, once or 2+ times
Burns	Had a burn severe enough to require professional medical attention? *(never; once; 2-5 times; 6-10 times; more than 10 times; prefer not to say)*	Never, once or 2+ times
Requires stiches	Required stitches for a cut? *(never; once; 2-5 times; 6-10 times; more than 10 times; prefer not to say)*	Never, once or 2+ times
