# Supplementary material for: Associations between Adverse Childhood Experiences (ACEs) and Lifetime Experience of Car Crashes and Burns: A Cross-Sectional Study
Source: Int J Environ Res Public Health. 2022 Nov 30;19(23):16036. doi: 10.3390/ijerph192316036 (PMC9735663; doi:10.3390/ijerph192316036)
Supplement: Supplementary file 1 [file ijerph-19-16036-s001.zip › Supplementary file S2 FINAL.pdf]

**Supplementary file S2**

**Table S1.** Bivariate relationships between injury outcomes and participant demographics

| | | | % Car crash | | | | % Burn[#] | | | |
|---|---|---|---|---|---|---|---|---|---|---|
| | | n | Ever | Never | Once | 2+ times | Ever | Never | Once | 2+ times |
| All | | 4783 | 52.6 | 47.4 | 24.4 | 28.2 | 11.6 | 88.4 | 9.6 | 2.1 |
| ACE count | 0 | 2412 | 48.5 | 51.5 | 23.4 | 25.2 | 9.0 | 91.0 | 7.9 | 1.1 |
| | 1 | 1056 | 52.9 | 47.1 | 24.3 | 28.6 | 11.3 | 88.7 | 9.2 | 2.1 |
| | 2-3 | 813 | 58.3 | 41.7 | 26.9 | 31.4 | 14.5 | 85.5 | 11.7 | 2.8 |
| | 4+ | 502 | 62.5 | 37.5 | 25.5 | 37.1 | 20.3 | 79.7 | 14.9 | 5.4 |
| | $X^2$ | | 46.462 | | | 52.62 | 59.888 | | | 72.658 |
| | p | | <0.001 | | | <0.001 | <0.001 | | | <0.001 |
| Gender | Male | 1809 | 63.0 | 37.0 | 23.9 | 39.1 | 13.2 | 86.8 | 10.4 | 2.8 |
| | Female | 2974 | 46.3 | 53.7 | 24.7 | 21.6 | 10.7 | 89.3 | 9.0 | 1.6 |
| | $X^2$ | | 125.580 | | | 189.21 | 7.135 | | | 10.03 |
| | p | | <0.001 | | | <0.001 | 0.008 | | | 0.007 |
| Age group | 18-29 | 448 | 39.5 | 60.5 | 25.4 | 14.1 | 16.3 | 83.7 | 11.8 | 4.5 |
| | 30-39 | 521 | 52.4 | 47.6 | 26.7 | 25.7 | 13.1 | 86.9 | 10.2 | 2.9 |
| | 40-49 | 787 | 63.0 | 37.0 | 23.9 | 39.1 | 12.5 | 87.5 | 10.2 | 2.3 |
| | 50-59 | 1037 | 58.4 | 41.6 | 23.9 | 34.5 | 12.1 | 87.9 | 9.9 | 2.1 |
| | 60-69 | 898 | 55.0 | 45.0 | 24.9 | 30.1 | 11.7 | 88.3 | 10.2 | 1.4 |
| | 70+ | 1092 | 43.2 | 56.8 | 23.4 | 19.9 | 8.0 | 92.0 | 7.0 | 1.0 |
| | $X^2$ | | 119.886 | | | 174.043 | 25.479 | | | 36.091 |
| | p | | <0.001 | | | <0.001 | <0.001 | | | <0.001 |
| Ethnicity | White | 4483 | 52.9 | 47.1 | 24.4 | 28.5 | 11.6 | 88.4 | 9.6 | 2.0 |
| | Other | 300 | 49.3 | 50.7 | 24.7 | 24.7 | 12.3 | 87.7 | 9.3 | 3.0 |
| | $X^2$ | | 1.408 | | | 2.185 | 0.157 | | | 1.373 |
| | p | | 0.235 | | | 0.335 | 0.692 | | | 0.503 |
| Deprivation quintile | 1 - most | 1204 | 47.8 | 52.2 | 23.5 | 24.3 | 15.6 | 84.4 | 11.9 | 3.7 |
| | 2 | 865 | 51.4 | 48.6 | 24.2 | 27.3 | 10.3 | 89.7 | 9.0 | 1.3 |
| | 3 | 847 | 51.4 | 48.6 | 23.1 | 28.2 | 13.0 | 87.0 | 10.5 | 2.5 |
| | 4 | 926 | 56.0 | 44.0 | 26.5 | 29.6 | 9.4 | 90.6 | 8.2 | 1.2 |
| | 5 - least | 941 | 57.8 | 42.2 | 25.0 | 32.8 | 8.7 | 91.3 | 7.5 | 1.2 |
| | $X^2$ | | 26.972 | | | 31.604 | 33.929 | | | 44.239 |
| | p | | <0.001 | | | <0.001 | <0.001 | | | <0.001 |
| Survey method | Telephone | 3944 | 52.8 | 47.2 | 23.7 | 29.0 | 10.8 | 89.2 | 9.1 | 1.7 |
| | Online | 839 | 52.1 | 47.9 | 27.7 | 24.4 | 15.4 | 84.6 | 11.7 | 3.7 |
| | $X^2$ | | 0.128 | | | 9.601 | 13.935 | | | 19.414 |
| | p | | 0.721 | | | 0.008 | <0.001 | | | <0.001 |
| Study area | Bolton | 1865 | 53.3 | 46.7 | 22.7 | 30.6 | 12.2 | 87.8 | 9.9 | 2.3 |
| | Wales | 2918 | 52.2 | 47.8 | 25.5 | 26.7 | 11.3 | 88.7 | 9.4 | 1.9 |
| | $X^2$ | | 0.523 | | | 9.751 | 0.891 | | | 1.235 |
| | p | | 0.470 | | | 0.008 | 0.345 | | | 0.539 |

ACE = adverse childhood experience.

**Table S2.** Logistic regression analysis of relationships between ACEs, socio-demographics, study area and survey type and having ever experienced injury types

| | | Car crash | | | | Burn[#] | | | |
|---|---|---|---|---|---|---|---|---|---|
| | | AOR | Low CI | High CI | P | AOR | Low CI | High CI | P |
| ACE count | 0 | Ref. | | | <0.001 | Ref. | | | <0.001 |
| | 1 | 1.22 | 1.05 | 1.41 | 0.011 | 1.22 | 0.96 | 1.55 | 0.101 |
| | 2-3 | 1.49 | 1.26 | 1.77 | <0.001 | 1.54 | 1.21 | 1.97 | 0.001 |
| | 4+ | 1.88 | 1.53 | 2.32 | <0.001 | 2.24 | 1.71 | 2.93 | <0.001 |
| Gender | Male | 2.13 | 1.88 | 2.41 | <0.001 | 1.35 | 1.13 | 1.63 | 0.001 |
| Age group | 18-29 | Ref. | | | <0.001 | Ref. | | | 0.059 |
| | 30-39 | 1.85 | 1.42 | 2.40 | <0.001 | 0.84 | 0.58 | 1.20 | 0.334 |
| | 40-49 | 2.79 | 2.17 | 3.57 | <0.001 | 0.84 | 0.60 | 1.19 | 0.327 |
| | 50-59 | 2.19 | 1.72 | 2.78 | <0.001 | 0.86 | 0.62 | 1.19 | 0.361 |
| | 60-69 | 1.86 | 1.46 | 2.38 | <0.001 | 0.85 | 0.61 | 1.20 | 0.367 |
| | 70+ | 1.15 | 0.91 | 1.47 | 0.245 | 0.59 | 0.41 | 0.84 | 0.003 |
| Ethnicity | White | 1.17 | 0.90 | 1.51 | 0.240 | 1.27 | 0.87 | 1.85 | 0.225 |
| Deprivation quintile | 1 - most | 0.62 | 0.52 | 0.75 | <0.001 | 1.79 | 1.34 | 2.38 | <0.001 |
| | 2 | 0.75 | 0.62 | 0.91 | 0.003 | 1.14 | 0.83 | 1.57 | 0.422 |
| | 3 | 0.77 | 0.64 | 0.94 | 0.009 | 1.54 | 1.13 | 2.09 | 0.006 |
| | 4 | 0.94 | 0.78 | 1.14 | 0.530 | 1.08 | 0.79 | 1.48 | 0.639 |
| | 5 - least | Ref. | | | <0.001 | Ref. | | | <0.001 |
| Survey method | Online | 0.99 | 0.84 | 1.16 | 0.899 | 1.34 | 1.07 | 1.68 | 0.011 |
| Study area | Wales | 0.88 | 0.77 | 1.00 | 0.045 | 0.93 | 0.77 | 1.13 | 0.469 |

AOR = adjusted odds ratio; CI = confidence interval (95%); Ref = reference category. Reference categories for gender, ethnicity, survey method and study area were female, other, telephone and Bolton Local Authority respectively. #Severe enough to require professional medical attention. Full wording of all questions is provided in Supplementary file S1.

**Table S3.** Multinomial logistic regression analysis of ACEs, socio-demographics, survey method and associations with car crashes and burns

| | | Car crash | | | | | | | | | Burn[#] | | | | | | | | |
|---|---|---|---|---|---|---|---|---|---|---|---|---|---|---|---|---|---|---|---|
| | | | Once | | | | 2+ times | | | | | Once | | | | 2+ times | | | |
| | | Reference category* | AOR | Low CI | High CI | P | AOR | Low CI | High CI | P | Reference category* | AOR | Low CI | High CI | P | AOR | Low CI | High CI | P |
| ACE count | 0 | <0.001 | Ref. | | | | Ref. | | | | <0.001 | Ref. | | | | Ref. | | | |
| | 1 | | 1.15 | 0.96 | 1.38 | 0.130 | 1.28 | 1.07 | 1.54 | 0.007 | | 1.15 | 0.89 | 1.49 | 0.292 | 1.74 | 0.98 | 3.08 | 0.060 |
| | 2-3 | | 1.43 | 1.17 | 1.75 | 0.001 | 1.54 | 1.26 | 1.89 | <0.001 | | 1.45 | 1.11 | 1.89 | 0.006 | 2.17 | 1.22 | 3.85 | 0.008 |
| | 4+ | | 1.53 | 1.18 | 1.97 | 0.001 | 2.30 | 1.80 | 2.93 | <0.001 | | 1.95 | 1.45 | 2.62 | <0.001 | 4.13 | 2.34 | 7.29 | <0.001 |
| Gender | Male | <0.001 | 1.47 | 1.26 | 1.71 | <0.001 | 3.01 | 2.59 | 3.49 | <0.001 | 0.001 | 1.25 | 1.03 | 1.53 | 0.028 | 1.98 | 1.32 | 2.98 | 0.001 |
| Age | 18-29 | <0.001 | Ref. | | | | Ref. | | | | 0.140 | Ref. | | | | Ref. | | | |
| | 30-39 | | 1.43 | 1.05 | 1.94 | 0.022 | 2.66 | 1.86 | 3.80 | <0.001 | | 0.88 | 0.58 | 1.32 | 0.531 | 0.73 | 0.36 | 1.48 | 0.386 |
| | 40-49 | | 1.68 | 1.26 | 2.26 | <0.001 | 4.95 | 3.55 | 6.90 | <0.001 | | 0.92 | 0.63 | 1.34 | 0.650 | 0.65 | 0.33 | 1.27 | 0.203 |
| | 50-59 | | 1.45 | 1.10 | 1.92 | 0.009 | 3.62 | 2.62 | 4.99 | <0.001 | | 0.93 | 0.64 | 1.34 | 0.690 | 0.67 | 0.35 | 1.29 | 0.226 |
| | 60-69 | | 1.40 | 1.05 | 1.86 | 0.021 | 2.76 | 1.98 | 3.85 | <0.001 | | 0.98 | 0.67 | 1.42 | 0.899 | 0.48 | 0.23 | 1.02 | 0.057 |
| | 70+ | | 1.05 | 0.79 | 1.39 | 0.746 | 1.40 | 1.00 | 1.96 | 0.049 | | 0.66 | 0.45 | 0.98 | 0.041 | 0.35 | 0.16 | 0.79 | 0.011 |
| Ethnicity | White | 0.227 | 1.05 | 0.77 | 1.42 | 0.781 | 1.31 | 0.95 | 1.80 | 0.095 | 0.447 | 1.28 | 0.83 | 1.95 | 0.264 | 1.27 | 0.60 | 2.70 | 0.532 |
| Deprivation quintile | 1 - most | | 0.73 | 0.59 | 0.92 | 0.007 | 0.53 | 0.42 | 0.66 | <0.001 | | 1.61 | 1.18 | 2.20 | 0.002 | 2.81 | 1.41 | 5.61 | 0.003 |
| | 2 | | 0.82 | 0.65 | 1.04 | 0.094 | 0.69 | 0.55 | 0.86 | 0.001 | | 1.17 | 0.83 | 1.64 | 0.364 | 0.97 | 0.42 | 2.27 | 0.948 |
| | 3 | | 0.79 | 0.63 | 1.01 | 0.056 | 0.75 | 0.60 | 0.95 | 0.016 | | 1.44 | 1.04 | 2.01 | 0.029 | 2.12 | 1.01 | 4.45 | 0.048 |
| | 4 | | 1.03 | 0.82 | 1.29 | 0.828 | 0.87 | 0.69 | 1.09 | 0.211 | | 1.09 | 0.78 | 1.53 | 0.620 | 1.01 | 0.43 | 2.35 | 0.982 |
| | 5 - least | <0.001 | Ref. | | | | Ref. | | | | <0.001 | Ref. | | | | Ref. | | | |
| Survey method | Online | 0.048 | 1.13 | 0.93 | 1.36 | 0.219 | 0.86 | 0.70 | 1.05 | 0.129 | 0.019 | 1.26 | 0.98 | 1.62 | 0.070 | 1.74 | 1.10 | 2.77 | 0.019 |
| Study area | Wales | 0.001 | 1.02 | 0.87 | 1.19 | 0.798 | 0.76 | 0.65 | 0.89 | 0.001 | 0.728 | 0.94 | 0.77 | 1.17 | 0.595 | 0.87 | 0.56 | 1.35 | 0.530 |

AOR = adjusted odds ratio; CI = confidence interval (95%); Ref = reference category. *Reference category for dependent variables was none. Reference categories for gender, ethnicity, survey method and study area were female, other, telephone and Bolton Local Authority respectively. [#]Requiring professional medical attention. Full wording of all questions is provided in Supplementary file S1.

**Table S4**. Multinomial logistic regression analysis of ACE types, socio-demographics, survey method and associations with car crash and burns

| | | Car crash | | | | | | | | | Burn[#] | | | | | | | | |
|---|---|---|---|---|---|---|---|---|---|---|---|---|---|---|---|---|---|---|---|
| | | | Once | | | | 2+ times | | | | | Once | | | | 2+ times | | | |
| | | Reference category* | AOR | Low CI | High CI | P | AOR | Low CI | High CI | P | Reference category* | AOR | Low CI | High CI | P | AOR | Low CI | High CI | P |
| ACE type | Physical abuse | 0.051 | 1.05 | 0.81 | 1.35 | 0.728 | 1.33 | 1.05 | 1.69 | 0.019 | 0.419 | 1.06 | 0.77 | 1.46 | 0.719 | 1.51 | 0.82 | 2.79 | 0.191 |
| | Emotional abuse | 0.012 | 1.06 | 0.85 | 1.32 | 0.605 | 1.37 | 1.11 | 1.69 | 0.004 | 0.157 | 0.83 | 0.62 | 1.12 | 0.218 | 0.62 | 0.34 | 1.15 | 0.129 |
| | Sexual abuse | 0.040 | 1.19 | 0.90 | 1.57 | 0.223 | 1.42 | 1.08 | 1.86 | 0.011 | 0.023 | 1.38 | 0.99 | 1.92 | 0.061 | 2.02 | 1.11 | 3.67 | 0.021 |
| | Divorce | 0.007 | 1.36 | 1.12 | 1.66 | 0.002 | 1.22 | 0.99 | 1.49 | 0.058 | 0.318 | 1.10 | 0.85 | 1.43 | 0.487 | 0.70 | 0.41 | 1.21 | 0.203 |
| | Domestic violence | 0.046 | 1.21 | 0.95 | 1.54 | 0.117 | 0.87 | 0.68 | 1.11 | 0.267 | 0.063 | 1.33 | 0.98 | 1.81 | 0.070 | 1.67 | 0.92 | 3.02 | 0.093 |
| | Mental illness | 0.189 | 0.85 | 0.67 | 1.07 | 0.167 | 1.07 | 0.85 | 1.34 | 0.579 | 0.016 | 1.47 | 1.11 | 1.94 | 0.007 | 1.50 | 0.86 | 2.63 | 0.157 |
| | Alcohol abuse | 0.814 | 1.08 | 0.83 | 1.41 | 0.564 | 1.07 | 0.82 | 1.39 | 0.621 | 0.429 | 1.08 | 0.77 | 1.50 | 0.661 | 1.49 | 0.81 | 2.73 | 0.202 |
| | Drug abuse | 0.778 | 1.12 | 0.71 | 1.78 | 0.629 | 1.17 | 0.75 | 1.83 | 0.497 | 0.346 | 0.78 | 0.45 | 1.36 | 0.386 | 1.55 | 0.69 | 3.49 | 0.287 |
| | Incarceration | 0.034 | 1.17 | 0.67 | 2.03 | 0.576 | 1.89 | 1.15 | 3.12 | 0.013 | 0.008 | 1.99 | 1.15 | 3.44 | 0.014 | 2.64 | 1.18 | 5.90 | 0.018 |
| Gender | Male | <0.001 | 1.45 | 1.24 | 1.69 | <0.001 | 3.07 | 2.64 | 3.58 | <0.001 | 0.001 | 1.21 | 0.98 | 1.49 | 0.075 | 2.13 | 1.37 | 3.30 | 0.001 |
| Age | 18-29 | <0.001 | Ref. | | | | Ref. | | | | 0.111 | Ref. | | | | Ref. | | | |
| | 30-39 | | 1.45 | 1.06 | 2.00 | 0.022 | 2.99 | 2.05 | 4.38 | <0.001 | | 1.01 | 0.65 | 1.57 | 0.961 | 0.89 | 0.42 | 1.90 | 0.762 |
| | 40-49 | | 1.69 | 1.24 | 2.30 | 0.001 | 5.36 | 3.75 | 7.65 | <0.001 | | 1.02 | 0.67 | 1.54 | 0.928 | 0.70 | 0.33 | 1.48 | 0.347 |
| | 50-59 | | 1.45 | 1.08 | 1.94 | 0.014 | 3.85 | 2.71 | 5.45 | <0.001 | | 1.06 | 0.71 | 1.58 | 0.782 | 0.69 | 0.33 | 1.44 | 0.316 |
| | 60-69 | | 1.41 | 1.05 | 1.91 | 0.024 | 2.94 | 2.05 | 4.20 | <0.001 | | 1.09 | 0.72 | 1.65 | 0.684 | 0.47 | 0.21 | 1.08 | 0.077 |
| | 70+ | | 1.04 | 0.78 | 1.40 | 0.780 | 1.46 | 1.02 | 2.10 | 0.041 | | 0.72 | 0.47 | 1.10 | 0.131 | 0.35 | 0.15 | 0.84 | 0.018 |
| Ethnicity | White | 0.411 | 1.05 | 0.76 | 1.45 | 0.775 | 1.25 | 0.90 | 1.74 | 0.191 | 0.486 | 1.24 | 0.78 | 1.95 | 0.364 | 1.41 | 0.61 | 3.30 | 0.423 |
| Deprivation quintile | 1 - most | | 0.71 | 0.57 | 0.90 | 0.004 | 0.51 | 0.40 | 0.64 | <0.001 | | 1.53 | 1.11 | 2.11 | 0.009 | 2.60 | 1.29 | 5.26 | 0.008 |
| | 2 | | 0.83 | 0.65 | 1.05 | 0.113 | 0.68 | 0.54 | 0.87 | 0.002 | | 1.14 | 0.81 | 1.62 | 0.451 | 0.78 | 0.32 | 1.91 | 0.584 |
| | 3 | | 0.79 | 0.62 | 1.00 | 0.050 | 0.76 | 0.60 | 0.95 | 0.019 | | 1.41 | 1.01 | 1.97 | 0.046 | 1.98 | 0.93 | 4.22 | 0.077 |
| | 4 | | 1.03 | 0.82 | 1.30 | 0.777 | 0.86 | 0.68 | 1.08 | 0.197 | | 1.09 | 0.77 | 1.53 | 0.634 | 0.96 | 0.41 | 2.25 | 0.923 |
| | 5 - least | <0.001 | Ref. | | | | Ref. | | | | 0.001 | Ref. | | | | Ref. | | | |
| Survey method | Online | 0.027 | 1.13 | 0.93 | 1.38 | 0.220 | 0.83 | 0.67 | 1.02 | 0.080 | 0.134 | 1.21 | 0.92 | 1.58 | 0.170 | 1.52 | 0.92 | 2.52 | 0.106 |
| Study area | Wales | 0.001 | 0.99 | 0.85 | 1.16 | 0.919 | 0.75 | 0.64 | 0.88 | <0.001 | 0.841 | 0.97 | 0.78 | 1.21 | 0.810 | 0.88 | 0.56 | 1.39 | 0.580 |

AOR = adjusted odds ratio; CI = confidence interval (95%); Ref = reference category. *Reference category for dependant variables was none. Reference categories for gender, ethnicity, survey method and study area were female, other, telephone and Bolton Local Authority respectively. [#]Requiring professional medical attention. Full wording of all questions is provided in Supplementary file S1.
